# Toxicological Risk Assessment of the Accidental Ingestion of a Honeybee (*Apis mellifera* L.) Present in Food

**DOI:** 10.3389/fvets.2020.583286

**Published:** 2020-09-30

**Authors:** Jéssica Baeça Rezende Marinho, Benito Soto-Blanco

**Affiliations:** Department of Veterinary Clinics and Surgery, Veterinary College, Universidade Federal de Minas Gerais (UFMG), Belo Horizonte, Brazil

**Keywords:** accidental ingestion, allergens, honeybee, toxicological hazard, venom, zootoxins

## Abstract

The aim of the present work was to evaluate the possible risk of toxic effects due to the ingestion of a honeybee (*Apis mellifera* L.) accidentally present in food. The methodology used in this study was a bibliographic survey of studies on the toxic effects related to honeybees, with a critical analysis of the possible risks of accidental ingestion of these insects. The amount of venom present in a bee is considered insufficient to induce detectable toxic effects in a person who ingests it by accident, and various components of the venom are destroyed by gastric secretions. However, despite the rare frequency, there is a risk of the ingestion of a bee, causing an allergic reaction to some components of the venom in sensitized individuals. In addition, pollen carried by a bee may cause an allergic reaction in a sensitive individual. Thus, the accidental ingestion of a bee present in a food does not pose the risk of toxic effects for the majority of the population but may promote allergic reactions in susceptible individuals.

## Introduction

**Figure 1:**
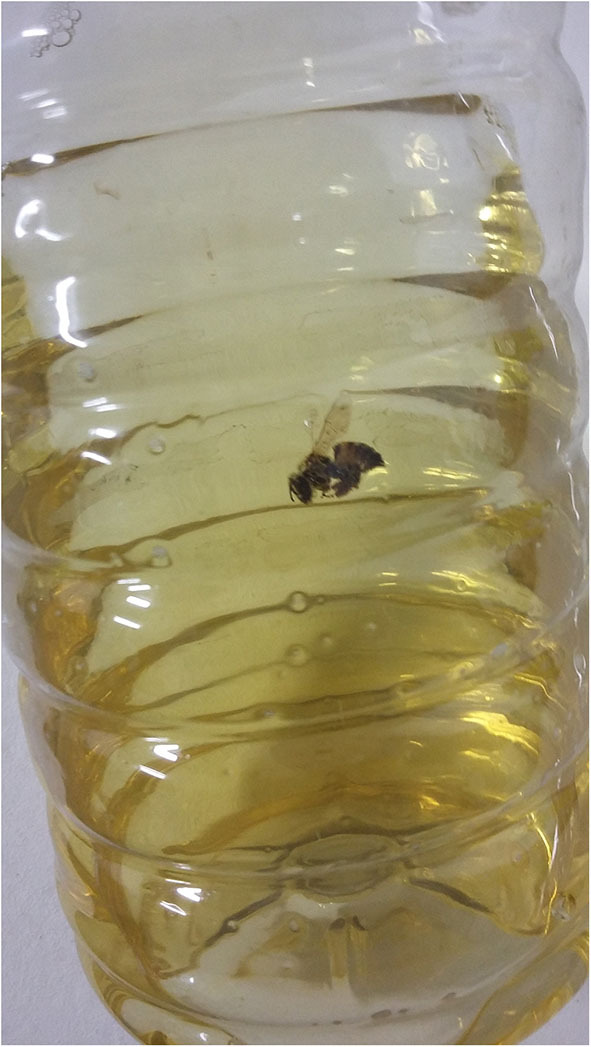
Honeybee trapped in an orange-flavored drink.

Honeybees (*Apis mellifera* L.) are social insects bred for the production of honey, pollen, propolis, royal jelly, wax, and poison, and to promote the pollination of various cultivated plant species. These bees have a sting as their defense mechanism, through which their venom is inoculated (1, 2). As honeybees seek products with sugar as their food source, they may end up trapped and incorporated into food (Figure 1), and potentially consumed inadvertently. A number of anecdotal reports of accidental ingestion of insects are largely available (3–6), but the actual number remains unknown.

The intentional consumption of insects by humans, known as entomophagy, is a habit of many populations around the world. Entomophagy has been advocated as a way to increase the availability of foods of recognized nutritional value (7). In the case of honeybees, they are traditionally eaten roasted or grilled in countries such as Japan, China, and Indonesia (8). Thus, it is possible that the heat used in the preparation methods may denature any harmful substances present.

However, there is a lack of information in the literature on the toxicological risks arising from the ingestion of raw honeybees. Thus, the aim of the present work was to evaluate the possible risk of toxic effects due to the ingestion of a honeybee accidentally present in food. The methodology used in this study was a bibliographic survey of studies on the toxic effects related to honeybees, with a critical analysis of the possible risks of accidental ingestion of these insects. The possibility that the ingested insect may carry a microorganism with the potential to cause infection was not addressed in this study.

## The Honeybee Venom

The honeybee sting is a defensive mechanism against predator attacks on individuals or hives (9–11). The stinger is a modification of the reproductive apparatus and is present only in worker honeybees. The venom, also known as apitoxin, is produced by venom gland cells and is injected at the time of the sting. When the stinger is introduced, it becomes trapped with the venom sac at the sting site and gradually releases the venom (10, 12, 13).

Honeybee venom is composed of peptides, including melittin, apamin, and mast cell degranulating peptide (MCDP), the enzymes phospholipase A_2_ and hyaluronidase, and biogenic amines (histamine, dopamine, and noradrenaline) (10, 12, 14, 15) (Table 1). Many other compounds were identified in the venom, but they are unlikely to possess toxicological importance.

**Table 1:** Main components of honeybee (*Apis mellifera*) venom (10, 12, 14, 15).

**Chemical groups**	**Compounds**
Peptides	Melittin
	Apamin
	Mast cell degranulating peptide (MCDP)
	Other peptides
Enzymes	Phospholipase A_2_
	Hyaluronidase
Biogenic amines	Histamine
	Serotonin
	Dopamine
	Noradrenaline

The peptide melittin is the most abundant toxin in honeybee venom, comprising ~40 to 60% of the dry weight of this venom, consisting of a basic 26-amino-acid polypeptide (12, 15, 16). Melittin monomers bind to lipid membranes producing pores, exerting a cytotoxic effect (17, 18). Furthermore, it acts synergistically with the enzyme phospholipase A_2_, promoting damage to the cellular and mitochondrial membranes of various cell types. Arachidonic acid may be released because of cell damage (10, 15). This peptide is probably the major responsible for the bee venom-induced pain through direct and indirect activation of primary nociceptor cells (16). As melittin has various pharmacological activities, including antitumoral (15, 19), anti-viral (18, 18, 20), antibacterial (15, 17), antifungal (15), anti-arthritis, anti-inflammatory, anti-atherosclerotic, anti-diabetic, and neuro-protective (12) effects, several studies have been conducted to evaluate the safety of the compound when administered orally. The results of these studies indicate that oral ingestion of this peptide results in low toxicity (21–23).

Apamin is a peptide neurotoxin comprising 2 to 3% of dry honeybee venom. This peptide is a specific inhibitor of small-conductance Ca^2+^-activated K^+^ (SK) channels in the central nervous system (13, 15) and activates the M_2_ inhibitory muscarinic receptors of the peripheral nervous system (10, 14, 15). Another activity is blockage of the Kv1.3 channel, a potassium channel type found in immune cells (24). Pharmacologically, apamin has antibacterial, antifungal, anti-inflammatory, anti-atherosclerotic, and antitumoral effects (13) and has been tested for treating neurological disturbances, including Parkinson's disease and learning deficit disorder (15).

The main enzymes present in the venom are phospholipases and hyaluronidase Phospholipase A_2_, comprises 10% to 12% of dry bee venom, promotes the disruption of the cytoplasmic membrane by the destruction of the constituent phospholipids, resulting in cell lysis (10, 14, 15, 25). This enzyme catalyzes the hydrolysis of glycerophospholipids, releasing fatty acids and lysophospholipids (25). P was also found to have antibacterial, trypanocidal, antitumoral, neuroprotective, and hepaprotective activities (15).

The other enzyme found in honeybee venom is hyaluronidase, which comprises 1 to 3% of venom. This enzyme promotes the fast tissue diffusion of venom through tissue disruption. Hyaluronidase causes the hydrolysis of hyaluronic acid in the extracellular matrix (10, 13–15). Other activities of hyaluronidase include mast cell degranulation and rise in blood vessel permeability (13).

Phospholipases and hyaluronidase are the allergenic proteins in the venom, being responsible for cases of anaphylactic reaction to honeybee venom (13, 15, 26). The enzyme phospholipase A_2_, isolated from microorganisms and vertebrate animals, is used in the processing of certain foods, and it has been experimentally verified that the consumption of phospholipase A_2_ residues does not represent a toxicological risk (27, 28). However, some cases of allergic reactions to honeybee phospholipase A_2_ residues present in honey consumed by sensitized individuals have been identified (29).

The peptide MCDP acts on mast cells, promoting degranulation releasing histamine, and consequent inflammation (13, 15). Paradoxically, large amounts of MCDP inhibits the release of histamine by mast cells (15). It also blocks fast activation and slowly inactivating K+ channels, resulting in neuronal hyperexcitability (30).

The biogenic amines present in honeybee venom are histamine, dopamine, and noradrenaline. Histamine, which amount present in the venom is smaller than that released by MCDP, promotes vasodilation enhancing the inflammation, whereas noradrenaline and dopamine have a well-known ionotropic effect (10, 13, 15). The ingestion of biogenic amines present in the honeybee venom sac probably does not represent a significant toxicological risk, because these amines are present in amounts that are high enough to impact human health (31, 32).

Honeybees can sting only once, leaving the stinger and venom sac in the sting site. A sting by one or a few honeybees promotes a reaction at the sting site that begins quickly and is characterized by pain, edema, and erythema. These local effects usually last for hours, but may, in some cases, continue for days. Multiple honeybee stings (a minimum of one hundred) are capable of promoting a systemic toxic reaction, characterized by agitation, vomiting, diarrhea, difficulty breathing, seizures, hyperthermia, and shock. Other clinical effects of the systemic toxic reaction are rhabdomyolysis and heart failure. In addition, sensitized individuals, who have been previously exposed to honeybee venom, may exhibit an anaphylactic reaction after only a single sting (33, 34).

A rare effect promoted is myocardial infarction, which usually occurs after multiple honeybee stings (35–38), but has also been observed as a result of only one sting (39). It is likely that myocardial infarction is caused by the spasm or thrombosis of the coronary arteries (39, 40) or is secondary to the hypersensitivity reaction (38).

It has been estimated that the amount of venom from honeybees that is lethal to 50% of humans by injection is 2.8 mg venom per kg of body weight. As a honeybee yields about 160 μg of venom (11), this amount is insufficient to cause detectable toxic effects in a person who has only ingested a single insect. In addition, as reported, various components of the venom are destroyed by gastric secretion. In addition, honeybee venom used for medical purposes is administered only by injection (41–43), rather than orally, most likely because it would lose its activity owing to degradation by the digestive system.

Remarkably, honeybee venom can cause allergic reactions in sensitized individuals (44–46). It has been found that even residual amounts of honeybee venom in honey can induce an allergic reaction, which is a very rare condition (29, 47). These allergic reactions are triggered mainly by the peptide melittin and the enzymes phospholipase A_2_, A_1_, and hyaluronidase. Allergic reactions to poison can be identified by the production of specific IgE and IgG4 antibodies in the serum of patients (48). Thus, although it is a rare condition, there is the risk that the ingestion of a honeybee may induce an allergic reaction to some venom components in sensitive individuals. In addition, the structural proteins of the honeybee itself may also induce an allergic reaction (47).

## Pollen

Honeybees are pollinating insects that collect pollen grains when visiting the flowers used for their food. The pollen grains collected by honeybees are agglutinated and transported to their colony in structures present in the hind legs named corbicles or pollen baskets. In addition, pollen grains also stick to the bee's body (49–51). In addition, to provide nutrients to bees, pollen serves as a source of enzymes that aid in the digestion of nutrients, such as beta-galactosidase, and helps to establish the beneficial digestive microbiota of these insects (52). For humans, pollen is a bee product known for its pharmacological activities, which include antimicrobial, anti-viral, anti-inflammatory, immunostimulatory, and antioxidant effects (53, 54).

The ingestion of pollen collected by honeybees may be responsible for the development of acute allergic reactions, including anaphylaxis, in sensitized individuals. Honeybee secretions probably do not significantly reduce the allergenic potential of the collected pollen (55). Although relatively rare, allergic reactions to pollen can be quite severe, even lethal (55–60). This reaction occurs after previous exposure to the compound that causes the reaction in the sensitized individual that usually does not occur at first exposure.

A large number of plant species may cause allergic reactions to pollen (Table 2). Thus, the pollen from several plant species that is collected by honeybees can cause allergic reactions in humans (60). Importantly, the same patient may have allergic reactions to pollen from more than one plant species simultaneously (58, 61, 62). In addition, patients who have allergic reactions to pollen may not be hypersensitive to honeybee venom components (61).

**Table 2:** Components of honeybee (*Apis mellifera*) venom.

**Common name**	**Scientific name**	**Plant family**	**References**
Chrysanthemum	*Chrysanthemum* spp.	Asteraceae	(61)
Dandelion	*Taraxacum* spp.	Asteraceae	(58, 61)
Mugwort	*Artemisia vulgaris*	Asteraceae	(55, 58)
Ragweed	*Ambrosia* spp.	Asteraceae	(61, 62)
Birch	*Betula* spp.	Betulaceae	(63, 64)
Arizona cypress	*Cupressus arizonica*	Cupressaceae	(58, 65, 66)
Japanese cedar	*Cryptomeria japonica*	Cupressaceae	(67)
Oak	*Quercus* spp.	Fagaceae	(68, 69)
Ash	*Fraxinus* spp.	Oleaceae	(69, 70)
Olive	*Olea* spp.	Oleaceae	(58, 71)
Privet	*Ligustrum* spp.	Oleaceae	(71)
Bahiagrass	*Paspalum notatum*	Poaceae	(71, 72)
Bermuda grass	*Cynodon dactylon*	Poaceae	(72, 73)
Corn	*Zea mays*	Poaceae	(73)
Johnson grass	*Sorghum halepense*	Poaceae	(73)
Reed canary grass	*Phalaris arundinacea*	Poaceae	(71)
Tall fescue	*Festuca arundinacea*	Poaceae	(71)
Ryegrass	*Lolium perenne*	Poaceae	(58, 62)
London planetree	*Platanus × acerifolia*	Platanus	(58)
Willows	*Salix* spp.	Salicaceae	(69)
Elms	*Ulmus* spp.	Ulmaceae	(69)
Nettle	*Urtica* spp.	Urticaceae	(69)

The allergic reactions to pollen occur after previous sensitization to their allergens. Pollen allergens are trapped and processed by dendritic cells that migrate to lymph nodes and induce the differentiation of naïve T helper cells into Th2 cells. The contact of epithelial barrier organs to pollen allergens can induce epithelial cells to release interleukin (IL) 25, IL-33 thymic stromal lymphopoietin. These factors re-activate Th2 cells that release IL-4, IL-5, and IL-9. IL-4 stimulates B cells to produce and release antigen-specific IgEs, whereas IL-5 activates eosinophils. Furthermore, IL-4 and IL-9 promote mast cell degranulation, releasing a number of compounds, including histamine, leukotrienes, cytokines, and chemotactic molecules, resulting in the clinical signs of allergic reaction (74).

A honeybee can carry more than 15 mg of pollen (75). In addition, pollen is also present within the digestive tract of a bee; a study evaluating two hives found that each honeybee contained, on average, 3.35 and 4.27 mg (76). It was found that one gram of pollen can contain between 400,000 and 6.4 million pollen grains (57). Patients exhibiting an allergic reaction after pollen consumption had a positive skin sensitivity test to 0.1 mg/mL pollen extracts (53, 54). Thus, pollen carried by a bee may cause an allergic reaction in a sensitized individual.

Pollen may contain toxic substances produced by plants (77–81), notably, pyrrolizidine alkaloids (82–86). These alkaloids have potent hepatotoxic effects and cytotoxic, genotoxic, and oncogenic activities; some compounds also have neurotoxic and nephrotoxic effects (87, 88). A study in Germany revealed that a total of 17 out of 55 pollen samples collected by honeybees and marketed in Europe contained detectable levels of pyrrolizidine alkaloids, with concentrations ranging from 1.08 to 16.35 μg/g (85). On the assumption of the volume of pollen carried by a honeybee as 15 mg (76), the amount of pyrrolizidine alkaloids ranging from 16.2 to 245.25 ng. As the ingestion of pyrrolizidine alkaloids up to 0.1 μg/kg per day is considered safe for humans (84, 86), the probable amounts found in a honeybee should not pose any toxicological risk.

Pollen may also contain pesticide residues used in agriculture (89–92). The highest residual pesticide concentration found in pollen collected by honeybees in a study conducted in the United States was 16.556 μg/g of the pesticide phosmet (89). Again, assuming the volume of pollen carried by a bee as 15 mg (75), this would be a maximum phosmet concentration of 248.34 ng, which would not pose a toxicological risk to humans as the acceptable daily intake for this compound has been set at 5 μg/kg body weight (93).

## Conclusions

The amount of venom present in a honeybee is considered insufficient to cause detectable toxic effects on a person who has accidentally ingested it; moreover, components of the honeybee venom are destroyed by gastric secretion. In contrast, despite the rarity, there is a risk of honeybee ingestion, causing an allergic reaction to some component of the venom in sensitized individuals. In addition, pollen carried by a honeybee may cause an allergic reaction in a sensitized individual. Thus, the accidental ingestion of a honeybee present in food does not carry a risk for the production of toxic effects for the majority of the population but may promote allergic reactions in susceptible individuals.

## Author Contributions

BS-B conceived the paper. JM and BS-B wrote the manuscript. All authors contributed to manuscript revision, read, and approved the submitted version.

## Conflict of Interest

The authors declare that the research was conducted in the absence of any commercial or financial relationships that could be construed as a potential conflict of interest.
